# GPU Accelerated Quantum Virtual Screening: Application for the Natural Inhibitors of New Dehli Metalloprotein (NDM-1)

**DOI:** 10.3389/fchem.2018.00564

**Published:** 2018-11-20

**Authors:** Mingsong Shi, Dingguo Xu, Jun Zeng

**Affiliations:** ^1^College of Chemistry, Sichuan University, Chengdu, China; ^2^MedChemSoft Solutions, Wheelers Hill, VIC, Australia

**Keywords:** virtual screening, QM/MM, GPU, NDM-1, natural inhibitors

## Abstract

Quantum mechanical approaches for the massive computation on large biological system such as virtual screening in drug design and development have presented a challenge to computational chemists for many years. In this study, we demonstrated that by taking advantage of rapid growth of GPU-based hardware and software (i.e., teraChem), it is feasible to perform virtual screening of a refined chemical library at quantum mechanical level in order to identify the lead compounds with improved accuracy, especially for the drug targets such as metalloproteins in which significant charge transfer and polarization occur amongst the metal ions and their coordinated amino acids. Our calculations predicted four nature compounds (i.e., Curcumin, Catechin, menthol, and Ferulic acid) as the suitable inhibitors for antibiotics resistance against New Delhi Metallo-β-lactamase-1 (NDM-1). Molecular orbitals (MOs) of the QM region of metal ions and their coordinated residues indicate that the bridged hydroxide ion delocalized the electron over the Zn-OH-Zn group at HOMO, different from MOs when the OH^−^ is not presented in NDM-1. This indicates that the bridged hydroxide ion plays an important role in the design of antibiotics and other inhibitors targeting the metalloproteins.

## Introduction

The drug discovery and development is an expensive and complex enterprise. The role of the computational chemist within this team has become firmly established as the computer-aided drug design impacts the development across the discovery time line, from target identification and hit discovery to hit-to-lead optimization and safety profiling (Loughney et al., 2011).

A common activity of the computational drug design is the prediction of the bound conformation of a ligand (“Docking”) or millions of ligands to a given receptor binding pocket (“virtual screening”). In general, widely used knowledge-based, empirical or force-field based scoring functions are capable of providing docked poses with reasonable accuracy. Along this line, development of virtual screening has been made in the areas of ligand-based methods including the field based methods (Jilek and Cramer, 2004), substructural descriptors (Vogt et al., 2010) etc., and of structure-based methods with many docking softwares available (see Sukumar and Das, 2011 and its references therein). More recently, the artificial intelligence (AI) and machine learning technologies have also been developed for virtual screening in drug discovery (Goh et al., 2017). However, these approaches are usually not applicable for the complicated pockets such as those with metal ions or highly polarizable sites (Wang et al., 2011). In these cases, the incorporation of the quantum mechanical calculations into the docking or virtual screening process (Illingworth et al., 2008) becomes essential for obtaining the reasonably accurate prediction which serves as the stepping-stone for further hit-to-lead optimization.

Over the last two decades, there has been a dramatic change in the computational power and accessibility of quantum mechanical software programs, enabling the application of quantum mechanical (QM) scheme to eve larger and more complex systems. It remains however that the highest level of QM can only be applied to modest system sizes of 10 s of atoms. A number of general strategies have been developed and employed to further extend the size of systems for drug-protein systems (Mucs and Bryce, 2013). The first is the QM/MM approach that describes a subregion of interest in electronic detail via QM and couples it to a larger environment modeled at the MM level. This approach has been widely used in study of enzyme activity (see review) (Senn and Thiel, 2009). The second approach is in the application of Fragment Molecular Orbital (FMO) (Fedorov et al., 2012). FMO scheme fragments the large molecules into small moieties as isolated monomers by using hybrid projection operators or adaptive frozen orbital. Non-additive terms can be recovered by considering combinations of dimers and trimers of the fragments. The third approach is the linear scaling methods (Zhou et al., 2010) which seek to reduce the scaling of computational cost order of N basis function. Several approaches have been developed by using localized molecular orbitals and divide-and-conquer method, as well as linear scaling wave plan technique. They have successfully applied to protein-ligand and protein-protein interactions at the QM level (Heady et al., 2006; Cole et al., 2010).

On the other hand, the rapid advances of computer power and technology brought up the importance of the utilization of computational architectures to accelerate the QM calculations. Distribution of the computational overheads of QM algorithms on parallel CPU-based architectures leads to tractable ab initio and density function QM calculations on thousands of basis functions (Valiev et al., 2010). Recently, work has focused on the adaption of QM algorithms to graphics processing units (GPUs) architectures. GPU-based approaches for DFT (Ufimtsev and Martinez, 2008, 2009a,b; Yasuda, 2008) and MP2 (Vogt et al., 2008) calculations have been developed and the software has also been advanced (Genovese et al., 2009; Ufimtsev and Martinez, 2009b; Asadchev and Gordon, 2012; Titov et al., 2013; Frisch et al., 2016).

This study emphasizes on the implementation of QM/MM approach into virtual screening for chemical libraries. By taking advantage of GPU-based architectures, we have developed a combined strategy to screen a library at QM level with a promising speed. To our knowledge, this is the first time to investigate the feasibility of GPU-based virtual screening of a chemical library at the QM/MM level.

We use New Delhi Metallo-β-lactamase-1 (NDM-1) as the example. The NDM-1 protein bearing Klebsiella pneumoniae and Escherichia coli infections, which have broad spectrum against all beta-lactams tested, were first discovered in India in 2008 (Yong et al., 2009). The occurrence of NDM-1 exaggerates the problem of bacteria resistance, and its rapid spreading also makes it become one of major threats to human health in recent years. However, no clinical effective inhibitors were reported so far. NDM-1 belongs to the family of β-lactamases, which can further be divided into A, B, C, and D subgroups. Basically, subfamily A, C, and D can be grouped into serine proteases, while subfamily B is a metalloprotein containing either one or two zinc ions to keep its activity. NDM-1 belongs to B1 family, in which two zinc ions have been identified with a hydroxide ion bridged between two zinc cofactors (Thomas et al., 2011). Based on the crystal structure NDM-1 complexed with captopril (King et al., 2012), the reaction mechanism of hydrolysis of ampicillin which leads to the drug resistance of bacteria has been studied both experimentally (Zhang and Hao, 2011) and theoretically (Zheng and Xu, 2013).

## Methodological details

### Model construction

**Figure 1:**
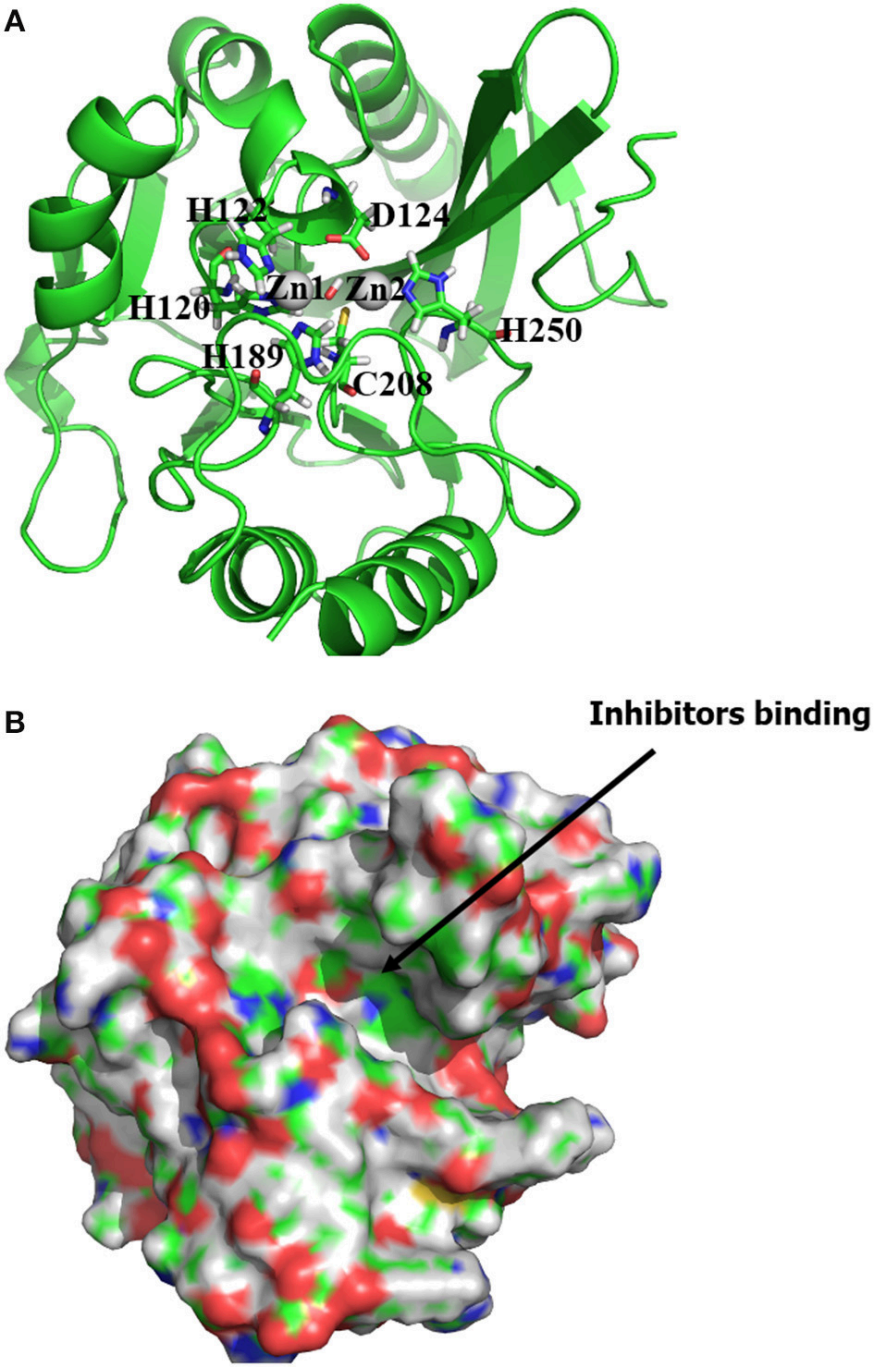
**(A)** Active binding sites of NDM-1 (PDB 3Q6X, Zhang and Hao, 2011), with the Zinc ions and the coordinated residues highlighted in sphere and stick, respectively; **(B)** surface presentation of the NDM-1. The general location for the ligand binding is arrowed. Figures were prepared using PyMol (Schrodinger, 2012).

The structure of NDM-1 was taken from crystal structure of the complex between NDM-1 and hydrolysed ampicilin (PDB 3Q6X, Zhang and Hao, 2011). Previous QM/MM study has suggested a hydroxide ion connecting two zinc cofactors for the hydrolysis of antibiotics and therefore been included in our previous model (Thomas et al., 2011; Zheng and Xu, 2013). Overall, the zinc ions are coordinated with residues (OH^−^, His120, His122, Asp123), and with residues (OH^−^, His189, Cys208, His250), respectively. The position of OH^−^ group was taken from previous study (Zheng and Xu, 2013), while the Cys208 is deprotonated with net charge of −1 as shown in Figure 1.

### Molecular mechanical virtual screening

Our docking program was used for virtual screening of the natural product library to the NDM-1 protein. The docking program consists of two-steps: (1) performing a multiple-copy simultaneous search “MCSS” (Zeng and Treutlein, 1999; Zeng, 2000) of common fragments such as benzene, pyridine, pyrimidine, pyrazine and phenol on the binding sites of a protein. The details have been described else (Zeng and Treutlein, 1999). In this study, we use the residue Glu123 as the center of the binding site. (2) docking a compound into the binding site of the protein. The fragments of functional groups were used as the guidance to place the compound inside the binding site and a torsional Monte Carlo minimization was performed to optimize the ligand conformation. Details of docking algorithm have been briefly described before (Stehn et al., 2013; Treutlein et al., 2017). Our software package quCBit (www.medchemsoft.com) has been developed and applied in this purpose. All-atom Charmm22 force field (MacKerell et al., 1998) was used and dielectric constant of 10 was used to screen the electrostatic interactions as implemented.

The docked conformations were selected based on its overlay criteria with the MCSS minima and the molecular mechanical interaction energies between the compound and protein. The overlay criterion is designated as following steps. Firstly, the sub-groups correspond to the fragment types within a compound. Secondly, the center of the sub-group of each docked conformation to the center of each MCSS minimum was calculated. If the distance is less than 2.0 Å, the sub-group is considered as overlay with MCSS minima. Only the docked conformations with all the sub-groups overlay were retained. By trial and error, interaction energy of 10.0 kcal/mol was used as threshold to select these conformations that will be subjected to further QM refinement.

### GPU-based QM refinement

A QM/MM scheme (Warshel and Levitt, 1976) was used for the QM refinement of all the docked conformations for each compound. In the scheme, the QM region contains residues two zinc ions, hydroxide ion, 6 residues (i.e., His120, His122, Asp124, His189, Cys208, and His250), as well as the compound. The remaining part is the MM region. Saturated hydrogen was added for the boundary between QM and MM regions (Field et al., 1990). The total binding energies of protein-compound in solutions were calculated as

(1) $$\begin{array}{r} \Delta E=E_{\text{complex}}^{PCM}-E_{protein}^{PCM}-E_{compound}^{PCM} \end{array}$$

where $E_{\text{complex}}^{PCM}$ and $E_{protein}^{PCM}$should be calculated using QM/MM for the complex and protein with Polarizable continuum model (PCM) to mimic the solvent. However, current version of teraChem cannot perform this type of calculations so that we use gas-phase calculation for complex and protein. As the protein atoms are fixed in both complex and protein during the calculations, the errors between the results of gas-phase and PCM could be minor. Therefore, the Equation (1) thus becomes

(2) $$\begin{array}{r} \Delta E=E_{complex}-E_{protein}-E_{compound}^{PCM} \end{array}$$

in which the *E*_*complex*_ and *E*_*protein*_ are the total energies of complex between protein and compound, and the total energy of the protein only, respectively. $E_{compound}^{PCM}$ is the energy of each compound in aqueous solution obtained using polarizable continuum model (PCM) (Barone et al., 1997) via Gaussian09 (Frisch et al., 2009) with dielectric constant of 80.0.

The structures of complex and NDM-1 protein were minimized using the combined QM/MM approach in which the QM is calculated using teraChem (Ufimtsev and Martinez, 2009b) and the MM region calculated using Amber12 (Case et al., 2012). Two hundred minimization steps were performed for each docked conformation obtained from section Molecular Mechanical Virtual Screening. During the minimization, DFT method with B3LYP functional (Lee et al., 1988; Becke, 1993) and 6-31G basis set (Ditchfield et al., 1971) was used for the QM region. We choose the standard basis set of 6-31G only as it significantly reduces the computational cost comparing with the 6-31G(d) with the minimum effect on the energy and structures of the QM region. For the MM region, Amber ff14SB force field (Maier et al., 2015) was used for all amino acids, and general atom force field (GAFF) (Wang et al., 2004) was applied for the compounds. All the teraChem/Amber calculations were carried out on GPU computers (GTX780) with two E3-1200 CPUs and all the Guassian09/Amber calculations were performed on two E5-2620 CPUs with 12 threads.

## Results and discussions

### Outcome of virtual screening

**Figure 2:**
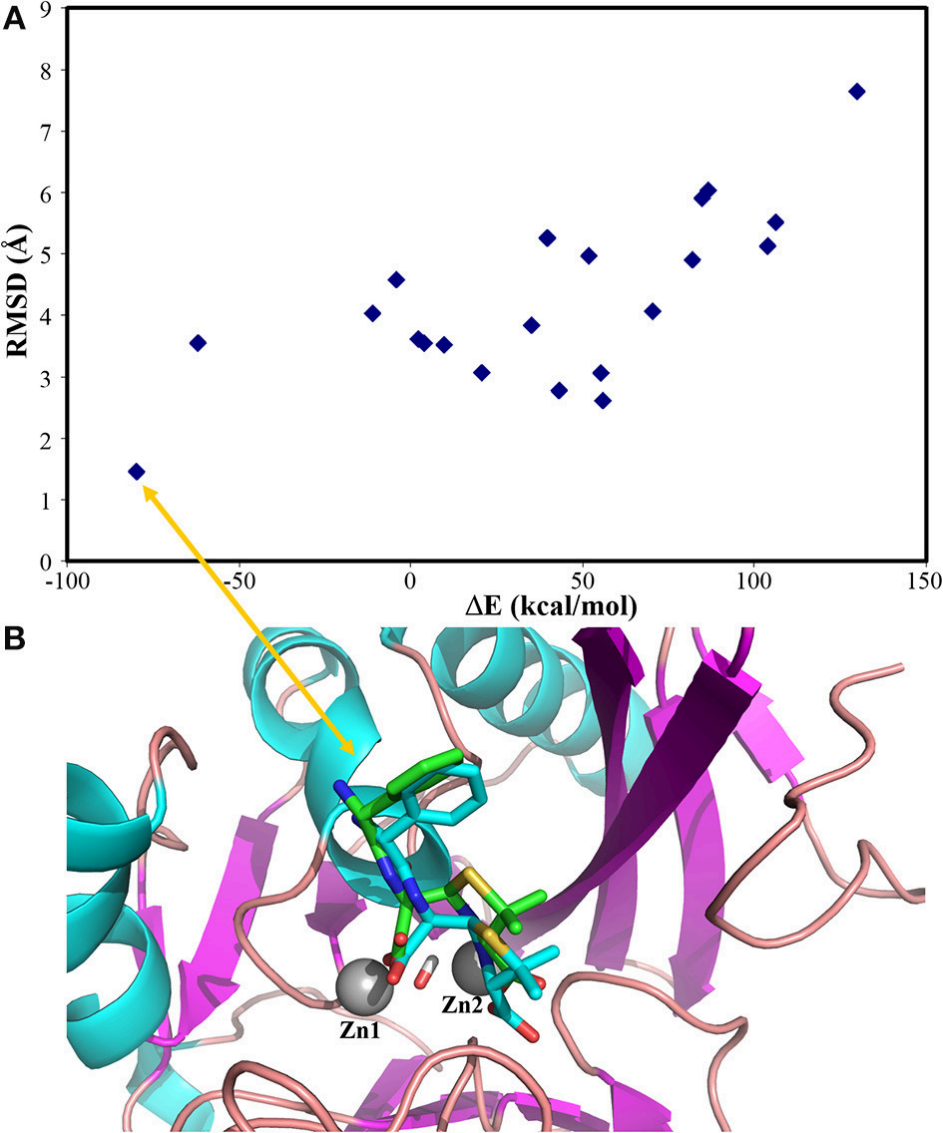
the predicted conformations of hydrolysed ampicillin with NDM-1 using the Docking/QM method vs. its crystal structure (PDB 3Q6X, Zhang and Hao, 2011). **(A)** Correlation of binding energies ΔE and the RMSD of the predicted conformations with the crystal structure; **(B)** the overlay the predicted structure with lowest DE (blue) with the crystal structure (green) from PDB 3Q6X (Zhang and Hao, 2011). Figure was prepared using PyMol (Schrodinger, 2012).

Our docking protocol was firstly used to predict the binding mode of hydrolysed ampicillin to the NDM-1, in comparison with crystal structure (PDB 3Q6X, Zhang and Hao, 2011). At first, our MM docking calculations give 21 conformations based on the score of protein-ligand interaction energies less than 10.00 kcal/mol and overlay with the MCSS minima as described in Method section Molecular Mechanical Virtual Screening. Using the GPU-accelerated QM refinement, these conformations were further optimized in the complex with NDM-1. Figure 2A plots the correlation between binding energies of docked conformations after QM refinement and its heavy atom RMSD to the crystal structure. Overall, the best conformation (shown in Figure 2B) of hydrolysed ampicillin with NDM-1 have the lowest binding energies of −79.82 kcal/mol with RMSD of 1.42 Å from the crystal structure. Based the QM protein-ligand binding energies Δ*E*, our combined approach of MM docking and GPU-accelerated QM refinement is capable to predict the native structure of ligand binding to the protein.

By applying our combined approach to screen the library of 34 nature compounds (Thakur et al., 2013), only 29 compounds were docked into the binding sites. Table 1 list the best binding energies of these compounds with NDM-1. Using the hydrolysed ampicillin as the reference (*E* = −79.82 kcal/mol), four compounds are considered as the potential inhibitors because they have value of *E* lower than the hydrolysed ampicillin. These hits include three compounds (Curcumin-Keto, Catechin, and Menthol) with strong binding energies of −84.25 kcal/mol, −83.76 kcal/mol, and −81.08 kcal/mol, and one compound (Ferulic acid) with equivalent binding energy of −79.12 kcal/mol, respectively.

**Table 1:** Calculated binding energies (ΔE) of nature compounds obtained using Equation (2).

**List**	**Molecules**	**Source**	**Formula**	**Number of atoms**	**Net charge**	**ΔE (kcal/mol)**
1	**Curcumin (keto form)**	***Curcuma longa***	**C**_21_**H**_20_**O**_6_	**47**	**0**	**–84.25**
2	Curcumin (Enol form)	*Curcuma longa*	C_21_H_20_O_6_	47	0	−14.74
3	Diosgenin	*Fenugreek*	C_27_H_42_O_3_	72	0	−43.90
4	Yamogenin	*Fenugreek*	C_27_H_42_O_3_	72	0	−50.18
5	Harmaline	*Peganum harmala*	C_13_H_16_N_2_O	32	0	−76.56
6	Harmine	*Peganum harmala*	C_13_H_14_N_2_O^2+^	30	+2	−11.20
7	Harmalol	*Peganum harmala*	C_12_H_12_N_2_O	27	0	−57.10
8	Tetrahydro-harmine	*Peganum harmala*	C_13_H_15_N_2_O^+^	31	+1	−71.07
9	Harmalan	*Peganum harmala*	C_12_H_12_N_2_	26	0	−72.04
10	Taraxerol	*Ciltorea Ternatea*	C_30_H_50_O	81	0	-
11	Rosmarinic acid	*Ocimum sanctum*	C_18_H_15_O${}_{8}^{-}$	41	−1	−42.96
12	Carvacrol	*Essential oils of oregano etc*.	C_10_H_14_O	25	0	−51.08
13	Caryophllene	*Essential oil of cloves etc*	C_15_H_24_	39	0	-
14	Zantrin, Z1		C_20_H_15_C_l3_O_3_	41	0	−48.76
15	Nimbolide	*Azadriachta indica*	C_27_H_30_O_7_	44	0	-
16	Gedunin	*Azadriachta indica*	C_28_H_34_O_7_	69	0	−29.01
17	Mahmoodin	*Azadriachta indica*	C_30_H_38_O_8_	76	0	−6.50
18	Margolone	*Azadriachta indica*	C_19_H_23_O${}_{3}^{-}$	45	−1	−56.88
19	Margonlonone	*Azadriachta indica*	C_19_H_21_O${}_{4}^{-}$	44	−1	−25.60
20	Isomargolonone	*Azadriachta indica*	C_19_H_21_O${}_{4}^{-}$	44	−1	−26.67
21	**Menthol**	***Mentha piperita***	**C**_10_**H**_20_**O**	**31**	**0**	**–81.08**
22	Campesterol	*Croton urucurana*	C_28_H_48_O	77	0	−66.43
23	**Catechin**	***Tea leaves***	**C**_15_**H**_14_**O**_6_	**35**	**0**	**–83.76**
24	Sonderianin	*Croton urucurana*	C_21_H_26_O_5_	52	0	−4.18
25	Stigmasterol	*Various plants and herbs*	C_29_H_48_O	78	0	−75.81
26	Gallocatechin	*Tea leaves*	C_15_H_14_O_7_	36	0	−75.30
27	Acetyl aleuritolic acid	*Euphorbiaceae*	C_32_H_49_O${}_{4}^{-}$	85	−1	-
28	**Ferulic acid**	***Scrophularia frutescense***	**C**_10_**H**_9_**O**${}_{4}^{-}$	**23**	**-1**	**–79.12**
29	Isovanillic acid		C_8_H_7_O${}_{4}^{-}$	19	−1	−34.79
30	Chrysin	*Tea leaves*	C_15_H_10_O_4_	29	0	−28.56
31	Carpaine	*Green plants and seeds*	C_28_H_50_N_2_O_4_	84	0	-
32	Berberine	*Barberry*	C_20_H_18_NO${}_{4}^{+}$	43	1	−24.11
33	Harmane	*Various of foods*	C_12_H_10_N_2_	24	0	−35.97
34	Warfarin	*Rubiaceae*	C_19_H_16_O_4_	39	0	−72.69

*The selected inhibitors are highlighted in bold*.

**Figure 3:**
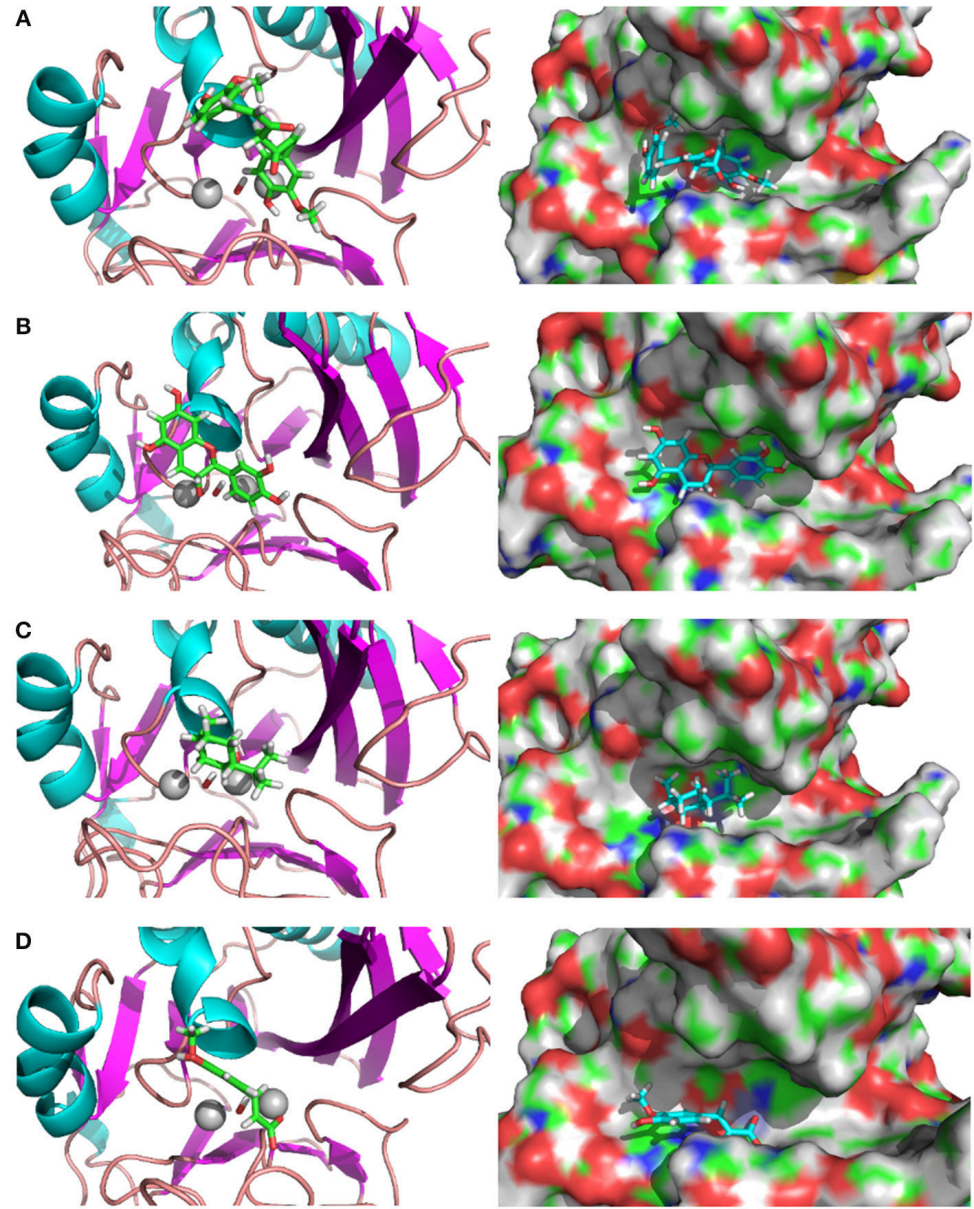
Binding structures of potential inhibitors with the NDM-1 and its location on the surface of NDM-1. **(A)** Keto form of curcumin; **(B)** catechin; **(C)** menthol; **(D)** Ferulic acid. Figures were prepared using PyMol (Schrodinger, 2012).

The complex structures of these inhibitors to NDM-1 are shown in Figure 3. The keto form of Curcumin has the strongest binding energy to NDM1 (−84.25 kcal/mol). The binding is dominated by interactions between the Zn-OH-Zn and the hydroxyl group of Curcumin. Similar feature is also found for Catechin and Menthol (Figures 3B,C). As shown in Figure 3D), Ferulic acid has the acidic group coordinated to Zinc ion directly; in addition, it also form electrostatic interactions to residue Lys393. This reminisces the binding structures of β-lactam antibiotics (e.g., ampicillin) to NDM-1 (Figure 2).

### MOs of divalent zinc ions with bridged hydroxide ion

**Figure 4:**
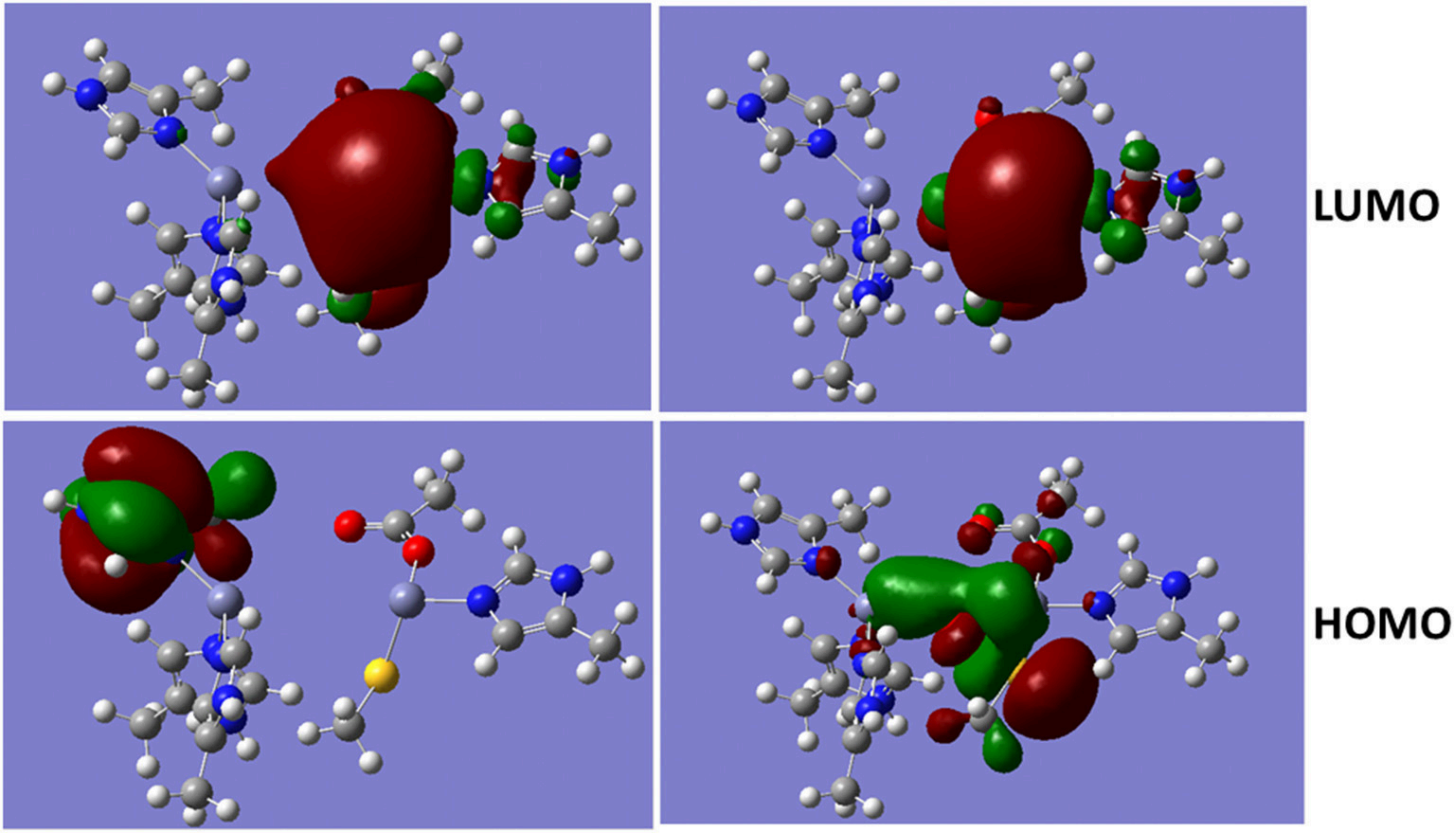
Molecular Orbital presentations (LUMO and HOMO) of QM region obtained using Guassian09. Left panel is NDM-1 without bridged hydroxide ion (PDB 4EXS, King et al., 2012) and Right Panel is NDM-1 in which a hydroxide ion is located between two Zinc ions (PDB 3Q6X, Zhang and Hao, 2011). Figures were prepared using Gaussview (Dennington et al., 2016).

The metalloproteins involved in bacteria infection have been a common target for the antibiotics. As a metalloprotein, NDM-1 can hydrolyse the antibiotics, and therefore, reduces or diminish its therapeutic effects. The hydrolysis reaction involves hydroxide ion between the two divalent Zinc ions (Thomas et al., 2011; Zheng and Xu, 2013). Figure 4 shows the Molecular Orbitals (MOs) of the QM region of the NDM-1 system with/without hydroxide ion. The MOs are obtained from the Gaussian09 calculations. While the LUMO are similar between two systems with the electron clouds concentrated around Zn2 ion, the OH^−^ bridge makes the HOMO delocalized over the Zn-OH-Zn cluster with a through-space π-π interactions between OH^−^ and S(–) of the deprotonated Cys208. Therefore, the inhibitors targeting on the NDM-1 will be of conjugate nature and interact Zn2 ion between deprotonated Cys208 and residue Gly211. This is consistent to the docked conformation predicted for the potential inhibitors of Curcumin-Keto, Catechin, Menthol, and Ferulic Acid as described above (Figure 3). In fact, the bridged hydroxide ions have been found in many metalloproteins, including oxygen transporters hemocyanin (Alzuet et al., 1997) and hemerythrin (Thomann et al., 1993), methane monoxygenase hydroxylse (Rosenzweig et al., 1993), and aminopeptidases (Bzymek et al., 2005) etc., and therefore, play critical role in the design of compounds that alter biological functions of metalloproteins.

### GPU vs. CPU performance

**Figure 5:**
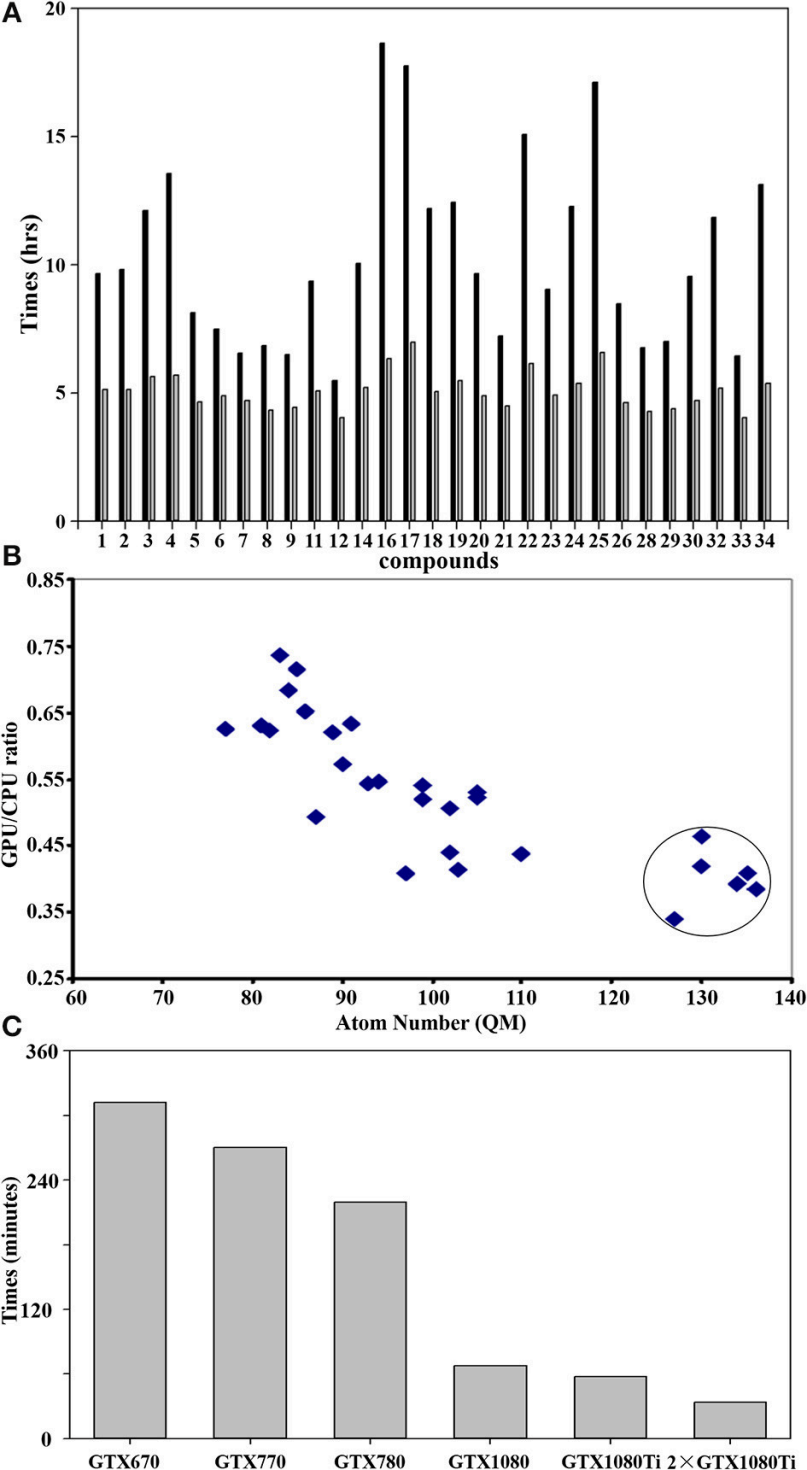
GPU accelerated performance of quantum mechanical calculations. **(A)** Comparison of performance of QM/MM optimization between CPU and GPU using Guassian09 and teraChem for the best docked conformation of the compounds as listed in Table 1. The x-axis sequence corresponds to the compound list in Table 1; **(B)** Correlation of GPU/CPU ratio (speedups) of the best docked conformation for each compound as function of atom numbers in the QM region. The speedups for the large size of QM region (>120 atoms) are circled. **(C)** Computer time (in minutes) required for a single QM calculation using teraChem as function of the GPU unit model. Currently, the most advanced GTX is GTX 1080ti.

Figure 5A shows the difference of QM calculations of a single docked conformation from each nature compound using GPU and CPU (12 threads) via teraChem and Guassian09, respectively. Overall, the GPU could significantly reduce the time cost up to 4-fold. The computational enhancement also depends on the size of molecules. As shown in Figure 5B, the significant reduction (up to 4-folds) of computational costs was achieved when the calculations were performed for the system with a QM region of more than 120 atoms.

GPU hardware also plays an important role in the performance of calculations. As shown in Figure 5C), GTX1080/1080ti speeded up the calculation up to 5-fold from GTX670. With two units of the most recent hardware GTX1080ti, maximal computer time cost for QM computation of a docked conformation can be reduced to less than 30 min, making the QM virtual screening of chemical library feasible.

It is noticeable that the computational time cost using GPU are consistent for each compound (Figure 5A), while the cost using CPU varies significantly according to its size. This is because the difference of the algorithm used for the computation of integral matrix between the teraChem and others. In order to make the full utilization of GPU architecture, tetraChem has optimized the computations of the integrals onto GPU units (Ufimtsev and Martinez, 2009b), and reduced certain of computation into single precision (Luehr et al., 2011).

## Conclusions

QM method has been used for analysis of protein-ligand interaction in drug design and discovery for many years. However, its expensive cost of computer power has hindered its application for virtual screening in drug design and development. Recent years, utilization of computational architecture to accelerate QM calculations has attracted significant attention (Furukawa et al., 2012; Ratcliff et al., 2017), and GPU-based approaches to perform QM calculations (Ufimtsev and Martinez, 2008) have been developed. Here, we have developed a combined strategy with docking the protein-ligand at MM level and re-rank the binding interaction at QM level. We choose the New Delhi metalloprotein 1(NDM-1) as the target due to its important metal binding sites in drug resistance and its significant threat to the world health (Johnson and Woodford, 2013).

With the GPU-accelerated QM refinement, our calculations have demonstrated that it is essential to re-rank the docked conformations at the Quantum mechanical level in order to identify the native binding conformation of a ligand close to the crystal structure, as demonstrated from the calculations of the hydrolysed antibiotic ampicillin to NDM-1 (Figure 2).

Our calculations predict potential inhibitors different from the previous docking study in which Isomargololone and Nimbolide were estimated to be the inhibitors of NDM-1 (Thakur et al., 2013). The discrepancy could be due to the factors that they used the crystal structure of NDM-1 (PDB 3Q6X, Zhang and Hao, 2011) as target without the bridged hydroxide ion and employed empirical docking and scoring function (Goodsell et al., 1996; Morris et al., 1998) only to estimate the binding affinities of the compounds. From our QM calculations, MOs of the QM region shows that the HOMO with the bridged OH^−^ between zinc ions has electron cloud delocalized over the Zn-OH-Zn cluster, while the LUMOs are similar with electron cloud concentrated at Zn2, independent of the hydroxide ion. As a result, the inhibitors of antibiotics resistance against the NDM-1 will be of conjugate nature, by binding over the Zn-OH-Zn, such as Curcumin, Catechin, and Feruclic acid (Figure 3).

The benchmark calculations presented here also provide some insight on the feasibility of virtual screening at quantum mechanical level. For a large QM region of more than 120 atoms which are very common for the metalloproteins, the GPU calculations can achieve the speedup of 4-fold comparing to the CPU method (Figure 5A). Previous study has demonstrated that 200-300 atoms would be needed as the minimum size of QM region in order to obtain accurate activation energies of enzyme reactions in proteins (Kulik et al., 2016). Moreover, the performance based on the GPU hardware infer that two units of the most advanced GPU architecture (GTX1080ti) could achieve the QM computation of single conformation within less than 30 min (Figure 5C). Furthermore, the speedup performance can be scaled up ca. twice by using two GPU processes, as demonstrated using two GTX1080ti units (Figure 5C).

Overall, our calculations demonstrated that it is feasible to use GPU-accelerated quantum mechanical method for virtual screening of chemical library. With the rapid advance of GPU hardware, quantum mechanical calculation in virtual screening will become more sophisticated and accurate at higher level. Here, we also showed that keto form of Curcumin, Catechin, Menthol, and Ferulic Acid may be considered as inhibitors for the antibiotics resistance against NDM-1. As natural product library is significantly smaller library than the synthetic chemicals, the approach proposed here will be of significance for accurate prediction of natural product hits as the lead compounds for the drug development.

## Author contributions

MS, DX, and JZ contributed conception and design of the study. MS organized the database. MS and DX performed the statistical analysis. MS wrote the first draft of the manuscript. DX and JZ wrote sections of the manuscript. All authors contributed to manuscript revision, read and approved the submitted version.

### Conflict of interest statement

The authors declare that the research was conducted in the absence of any commercial or financial relationships that could be construed as a potential conflict of interest.
